# Auditory feedback effect on temporal patterns during self-pacing treadmill walking

**DOI:** 10.1371/journal.pone.0335971

**Published:** 2025-11-04

**Authors:** Trevor V. Evans, Megan E. Reissman, Timothy Reissman, Mark Shelhamer, Ajit M. W. Chaudhari

**Affiliations:** 1 Department of Mechanical and Aerospace Engineering, The Ohio State University, Columbus, Ohio, United States of America; 2 Department of Mechanical and Aerospace Engineering, University of Dayton, Dayton, Ohio, United States of America; 3 Johns Hopkins University School of Medicine, Baltimore, Maryland, United States of America; 4 School of Health and Rehabilitation Sciences, College of Medicine, The Ohio State University Wexner Medical Center, Columbus, Ohio, United States of America; 5 Department of Biomedical Engineering, The Ohio State University, Columbus, Ohio, United States of America; ASPIRE Academy for Sports Excellence, QATAR

## Abstract

Self-pacing treadmills provide advantages for assessing locomotion such as having a controlled environment and ability to accurately collect prolonged data, but the variable sounds from the treadmill belt motors when changing speed could provide artificial sensory feedback to walkers that may influence their gait while on the self-pacing treadmill. We hypothesized that temporal measures of gait on a proportionally-controlled self-pacing treadmill would be significantly different between when sound is present vs removed. Participants (n = 31) walked under two different conditions for five-minute periods each on the self-pacing treadmill: one without headphones, and the other with noise-cancelling headphones playing brown noise to mask the treadmill motor sounds. Mixed effects models were used to assess the impact of condition on temporal gait patterns. A custom accelerometer-based algorithm was created to detect gait events while on the treadmill. Significant differences were found in the average values of swing time, step time, and gait speed between the treadmill conditions. These differences between the two treadmill conditions suggest that self-pacing treadmill walkers may utilize the variable belt motor sounds available to them. Given the potential incorporation of auditory feedback for motor planning when walking on the self-pacing treadmill, researchers should consider belt motor sounds as a potential factor that affects gait patterns.

## Introduction

### Overground vs treadmill walking

Treadmills are frequently utilized in research for gait analysis to simulate overground locomotion [1–3]. They offer several advantages including: controlled indoor environments, convenience to walk in a relatively small space, and ability to collect very accurate biomechanical data over long time periods. Standard treadmills operate at a single fixed speed that is pre-determined before the belts begin to move or require manual input to change the belt speed. Walking on a fixed-speed treadmill (FST) does not allow for spontaneous speed fluctuations, which we know occur in standard overground walking [4,5]. Holding a key aspect such as gait speed constant also likely affects spatiotemporal parameters such as stride time and stride length which are directly related to speed [6]. This limits the generalization of FST walking research to human locomotion. Other studies comparing overground to FST walking have found differences in kinematic, spatiotemporal, and kinetic parameters [7].

The development of a self-pacing treadmill (SPT) enables continuously adjusting belt speed so that researchers can retain the advantages of FST without predetermining a fixed speed [8,9]. Several feedback control approaches for SPT have been identified, including position-based [8,10–14], behavior-based [15], and force-based [16–20] algorithms. Only a few studies have compared SPT and overground walking paradigms in healthy adult populations [10,11,21], which is an important validation to support future research using SPT-based gait analysis in populations with medical conditions. Gait speed, often regarded as the most important measure of gait, was not found to differ significantly between SPT and overground walking [10,21]. Spatiotemporal measures show mixed findings since one study reported differences in swing and stance, single and double support, and step width among others [21], while another study reported no difference among any of the variables [11]. Both study protocols used position-based feedback control and collected overground data in similar ways, so there remains a need to further research on why spatiotemporal differences may exist between the two walking paradigms.

### Role of sound in movement

The large motors that actuate SPT belts produce continuous auditory noise in virtually all SPT systems, regardless of whether belt velocity is changing. The auditory noise profile changes at variable intensity and frequency levels when the belts change velocities. The exact auditory characteristics change based on the hardware design of the treadmill. However, these sounds are perceptible stimuli for the person walking on the SPT, who is constantly using several sensory feedback modalities to maintain their balance during walking [22,23].

Hearing allows us to perceive sound information from our environment, and we incorporate auditory stimuli among our other senses to aid in making goal-oriented decisions [24,25]. In walking, we use hearing to receive spatial and temporal cues that may help us better control our balance [26]. External perceivable auditory cues have been shown to alter gait performance in people who are healthy [27,28] and those with Parkinson’s disease [29,30]. One study reduced auditory feedback during gait using white noise in older adults, but found no differences in spatial or temporal aspects of walking [31]. However, they used a FST which does not have the variable sound profile that a SPT does. When this feedback is removed, temporal parameters from this SPT paradigm could be closer to measurements observed during overground walking.

### Purpose

The purpose of this study was to evaluate the effect of audible treadmill motor belt sounds on self-pacing treadmill temporal walking patterns and gait speed. We hypothesized that significant differences in stride, step, swing, and stance time measures would exist between conditions where participants could hear the variable sounds from the self-pacing treadmill belt motors versus when they could not. Furthermore, we hypothesized that there would be significant differences in gait speed between the two self-pacing treadmill conditions.

## Methods

### Participant recruitment

All study procedures were approved by The Ohio State University Institutional Review Board (IRB#2019H0188). Those who met the inclusion criteria were enrolled in the study as participants after providing institutional review board–approved written informed consent. Healthy adult participants within the age range 18–55 in the local area surrounding The Ohio State University were recruited for this study. Recruitment began on the 29^th^ of March, 2022 and lasted until the 19^th^ of February, 2024. Paper flyers and email listservs served as the means for recruitment. Participants who self-reported that they were unable to walk for an hour consecutively, or that they had experienced a diagnosed lower body injury within the past three months before the date of collection, were excluded from the study.

### Experimental protocol

The protocol outlined and data collected here were a part of a larger study. Wireless tri-axial accelerometer sensors (Trigno Legacy, Delsys Inc.; Natick, MA) were placed at three locations along each participant’s body: one directly below each of the left and right medial tibial condyles on the shank, and one over the sacrum. The leg sensors were secured using wraps with hook-and-loop fasteners, and the sacral sensor was placed in a pouch inside of a belt made of hook-and-loop fabric around the waist.

The self-pacing treadmill’s controller used an optical tracking system to keep the participant in the center of the treadmill. A lower back cluster of four reflective markers was attached over the sacrum to the waist belt, so it sat directly over the sacrum accelerometer. The position of the centroid of the sacrum marker cluster was used to determine the walker’s fore-aft position along the treadmill. The treadmill’s belt speed was increased or decreased proportionally to the cluster’s (walker’s) distance away from the treadmill midpoint (Supplemental Material, S1 Fig). Participants were attached to an overhead safety harness on an instrumented split-belt treadmill with right and left belts (Immersive Lab; Bertec Corp.; Columbus, OH), given a brief explanation of how a SPT functions differently from a fixed-speed treadmill, and instructed to walk at their preferred speed for the duration of the protocol. The two belts always moved at the same speed. Participants began to walk for an acclimation period of at least five minutes, after which they were asked to confirm that they felt comfortable with walking on the SPT. Following the acclimation period, a five-minute baseline SPT recording period of accelerometer data began as participants walked while hearing all environmental sounds (Supplemental Material, S1 File).

Participants were then given noise-cancelling wireless headphones (Model QuietComfort 35; Bose Corp., Framingham, MA) to remove perceived audible treadmill belt motor sounds from the participant while walking. Brown noise [32] was played through the headphones, with the volume increased incrementally until participants confirmed they could not hear any environmental sounds. Accelerometer data from one five-minute SPT walking trial with headphones worn were collected.

The self-pacing treadmill controller used for both treadmill walking trials was created and executed in MotionMonitor xGen (Version 3.57; Innovative Sports Training; Chicago, IL). MotionMonitor’s built-in proportional feedback controller was used to adjust the belt speed based on the anterior-posterior position of the lower back marker cluster relative to the center of the treadmill. Marker trajectories were tracked in real-time using MotionMonitor connected to a nine-camera Vicon motion capture setup, which acquired data at 100 Hz (Tracker Software, Vero Cameras; Vicon Motion Systems Ltd; Oxford, UK). Belt speed was adjusted by the controller at 100 Hz.

Three-axis accelerometer data from both walking trials (treadmill, treadmill with sound removal) of synchronized sensors placed on the left shank, right shank, and pelvis were captured at 148 Hz and saved in MotionMonitor. Accelerometer data were used for event detection. Vertical ground reaction force data recorded at 1000 Hz from the treadmill walking trials were also captured and saved in MotionMonitor to use for validation of accelerometer-based gait event detection. The original times of each recorded sample for accelerometer and GRF data were retained in the analysis, without any linear interpolation.

Accelerometer data and ground reaction force data were analyzed in MATLAB using custom scripts (Version 2023b; MathWorks, Inc.; Natick, MA).

### Accelerometer-based Gait event detection algorithm development and validation

Gait event detection is important for identifying events used to characterize human walking, particularly heel-strike and toe-off times. Wearable inertial sensors have recently been favored due to their compact and inexpensive designs, and event detection using them shows low error in comparison to the standard force plate and foot switch equipment [33–35]. Therefore, these sensors would be appropriate to assess gait events consistently during both treadmill and overground walking. However, there is a lack of data in the literature validating event detection algorithms used for overground walking. Moreover, accelerometer-based event detection can be advantageous on a side-by-side split-belt treadmill as well because of the loss of clean ground reaction force data when the participant steps simultaneously on both belts.

**Fig 1:**
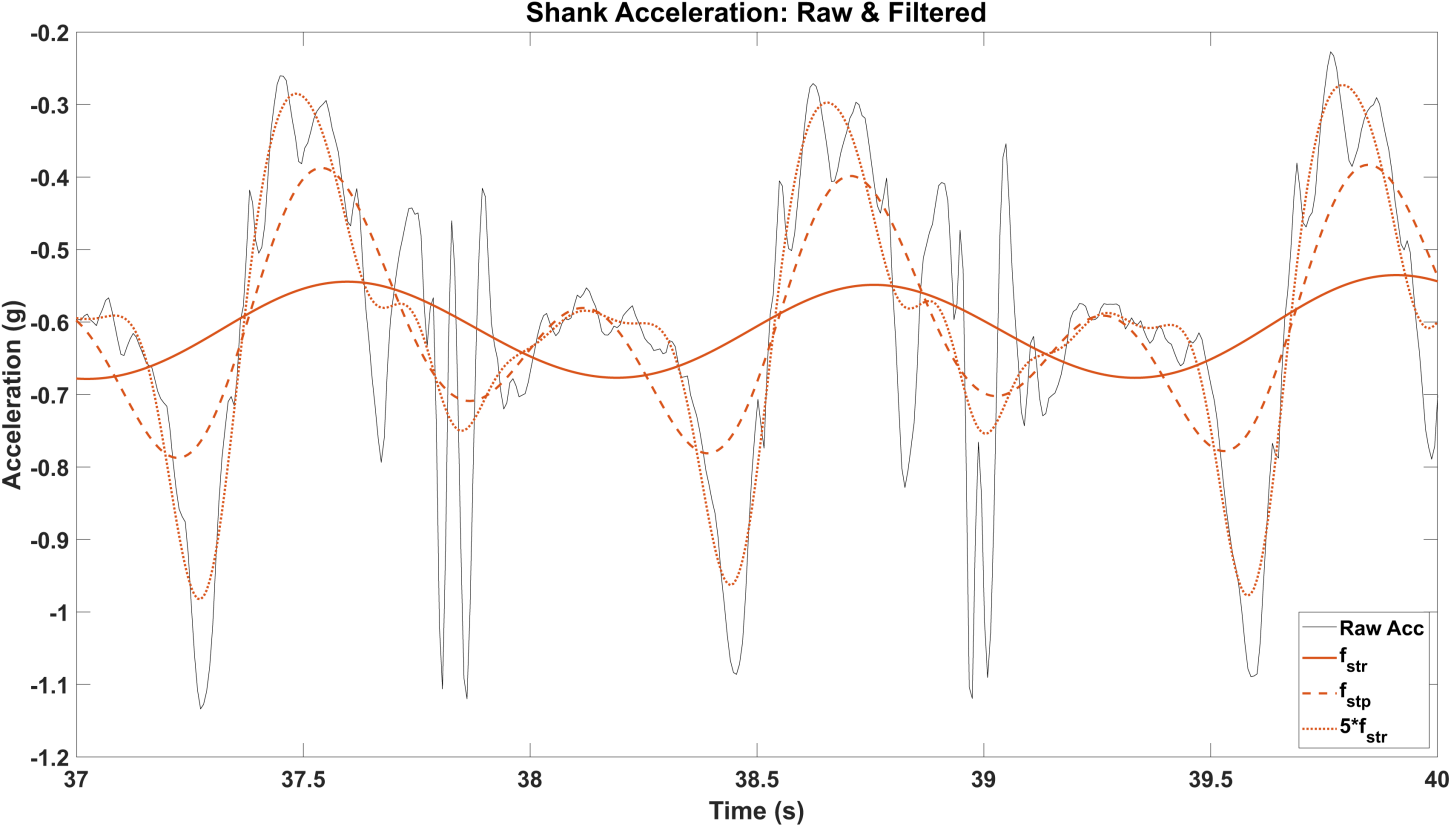
Shank acceleration: Raw & filtered. Raw long-axis shank acceleration (black solid) and shank acceleration low-pass filtered at three frequencies: stride frequency (f_str_; solid orange), step frequency (f_stp_; thick dashed orange), and 5 * stride frequency (5 * f_str_; thin dashed orange) of baseline.

The algorithm we used to identify gait events is modified from a previous study that detected gait events from thigh-worn accelerometers [35]. Gait events here were calculated from accelerometer data of the sensors placed on the medial left and right shanks. Left heel-strike and toe-off events were found using data from the accelerometer aligned with the long axis of the left shank, which was oriented vertically when participants stood in the anatomical position. First, single-sided power spectral densities (PSD) of the accelerometer signal were estimated using fast Fourier transformations. A moving average filter with window size of 50 samples was applied to the PSD data to reduce noise and identify the two largest frequencies in the signal. The frequency 10% above the largest peak between 0.6–1.4 Hz was estimated as the stride cutoff frequency (f_str, L_). The frequency 10% above the largest peak in the range of 1.4–2.5 Hz was estimated as the step cutoff frequency (f_stp, L_). The accelerometer signal was then low-pass filtered at three cutoff frequencies: f_str,L_, f_stp,L_, and 5 * f_str,L_, with the last of the frequencies being chosen to remove noise while maintaining fidelity of the true movement data. Using the stride frequency to estimate the largest cutoff frequency allows for participant-specific adaptability to a range of gait speeds. These three filtered accelerometer signals (Fig 1) were used to estimate gait events for the left shank, and the same process was performed independently for the right shank.

**Fig 2:**
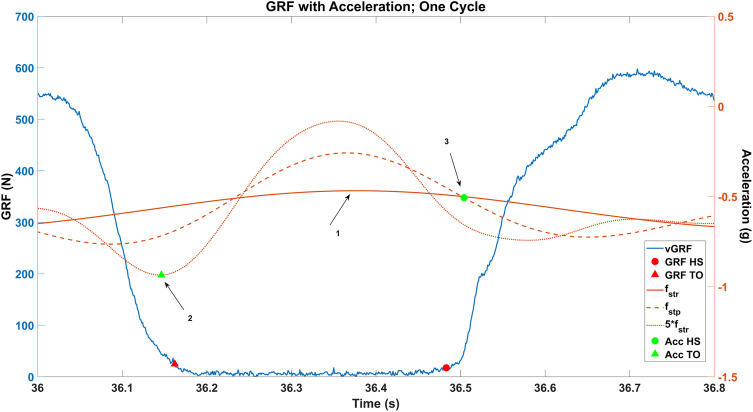
vGRF and filtered acceleration signals with detected gait events. Arrow 1: maximum values of the f_str_ signal; Arrow 2 – The time at which the minimum value of the 5 * f_str_ signal directly preceding each f_str_ maximum is estimated as the toe-off event (green triangle, Acc TO); Arrow 3 – Following each f_str_ maximum, the intersection of the f_str_ and f_stp_ curve is estimated as the heel-strike event (green circle, Acc HS). A 20 N threshold was used to identify the ground-truth heel-strike (red circle, GRF HS) and toe-off events (red triangle, GRF TO) from the vertical ground reaction force (vGRF). GRF HS = ground reaction force heelstrike; GRF TO = ground reaction force toe-off; f_str_ = stride frequency; f_stp_ = step frequency.

To identify gait events from accelerometer signal features, all maxima of the f_str_ -filtered accelerometer signal were first identified (Fig 2, Arrow 1), which were general indicators of the ipsilateral leg being in the mid-swing phase. Next, the minimum value of the 5 * f_str_ signal directly preceding each f_str_ maximum was estimated as the toe-off event (green triangle) (Fig 2, Arrow 2). For heel-strike identification, the succeeding crossing of the f_str_ -filtered signal with the f_stp_ -filtered signal after each f_str_ maximum was identified and chosen as the estimate (Fig 2, Arrow 3). This process was repeated for the right shank accelerometer data to detect gait events.

To validate this event-detection algorithm, vertical ground reaction force (GRF) data from the same SPT treadmill walking with sound trials were used as baseline reference values. A 20 N threshold was used to identify the heel-strike and toe-off events (Fig 2). Positive crossings were identified as heel-strike events, and negative crossings were identified as toe-off events.

Twelve participants with clean vGRF data were used for the validation of the accelerometer event detection algorithm out of the total 34 who enrolled in the study. Validation was performed based on the five-minute duration initial SPT walking condition with sound. Twenty-one participants were excluded from the validation analysis because they placed both feet on the same belt for a significant portion of at least one of the treadmill walking trials, resulting in no usable GRF data. Another participant was excluded from both the algorithm validation and testing of the primary hypothesis due to difficulties with collecting accelerometer data.

Accuracy of the gait event detection algorithm was quantified in MATLAB by calculating the signed and absolute errors of the accelerometer-based timing estimates from the reference GRF-based times of heel-strike and toe-off events (Table 1). The average signed errors of the accelerometer-based estimates (Table 1, row 2) across all participants were treated as systematic bias in the gait detection algorithm and were applied as corrections to the final estimates. After correcting for this systematic bias, the average absolute error was calculated again, and this correction reduced the absolute error of the final estimates (Table 1, row 3).

**Table 1:** Event detection error differences between gait events detected from shank accelerometers and force plates. The initial estimates were based on peaks and crossings of the filtered shank accelerometer data (rows 1 & 2). The systematic biases were negative indicating that the initial accelerometer-based estimates preceded the GRF-based estimates. Removing the systematic bias reduced absolute error in the final estimates of heel strike and toe off (row 3). SD: standard deviation of the average errors.

		Gait Event			
		Heel Strike		Toe Off	
		Left	Right	Left	Right
Initial Estimate	Abs. Error ± SD (ms)	26.1 ± 6.6	31.8 ± 8.2	21.4 ± 11.3	18.7 ± 7.3
Systematic Bias of Initial Estimate	Signed Error ± SD (ms)	−23.9 ± 14.3	−27.9 ± 17.7	−17.0 ± 17.5	−16.2 ± 14.1
Final Estimate	Abs. Error ± SD (ms)	14.1 ± 6.6	16.2 ± 8.2	14.4 ± 11.3	12.7 ± 7.3

### Statistical analysis

Mixed models for repeated measures were used to test the hypotheses for this study. Walking paradigm as a nominal variable was chosen as the fixed effect (SPT with sound vs SPT with sound removed) and participants as a random effect. The models were used to test for significant differences in the averages of temporal parameters including: stride time, step time, swing time, stance time, and gait speed (JMP Student 18.2; JMP Statistical Discovery LLC; Cary, NC). Step time, swing time, and stance time were averaged across both legs. Cohen’s d effect sizes were estimated for significant differences using least square means, standard errors, and denominator degrees of freedom. Gait speed was included as a covariate for all parameters since it likely varies across participants. Treadmill belt speed was used as a proxy for gait speed. The significance level was set *a priori* at α = 0.05. Normality assumptions were verified for the difference between walking paradigms for each parameter using Shapiro-Wilk tests (all p > 0.05).

## Results

Participants (n = 34) were enrolled in the study that satisfied the inclusion criteria. As mentioned above, data from 12 of these participants (4 M, 8 F; Age: 22.7 ± 4.16 yrs.) were used to validate the accelerometer event detection algorithm by comparing it to reference ground reaction force data. Data from the remaining participants were excluded from use in the validation due to quality control issues.

### Accelerometer event detection algorithm validation

Table 1 summarizes the errors in the accelerometer-detected gait events compared to reference values from the ground reaction force data. An average of 532 steps (standard deviation: 27.72 steps; range: 488–590 steps) from each participant during their five-minute baseline SPT walking trial were used to validate the algorithm. Initial estimates of mean absolute error ranged from 18.7–31.8ms, and systematic biases of 16.2–27.9ms were identified, with the accelerometer-detected events occurring before the GRF-detected events. Removing the mean errors from every event estimate led to final mean absolute errors from 12.7–16.2ms.

### Walking paradigm comparison

Data from 31 participants (17 M, 14 F; Age: 21.7 ± 3.1 yrs.) were used to compare the two walking paradigms (SPT with sound, SPT with sound removed) after eliminating data from three participants (n = 1 stopped walking during one of the trials, n = 2 had unusable accelerometer data for 1 leg). No asymmetries were observed across participants in any measures between the left and right legs, so all data were collapsed for further analyses.

**Fig 3:**
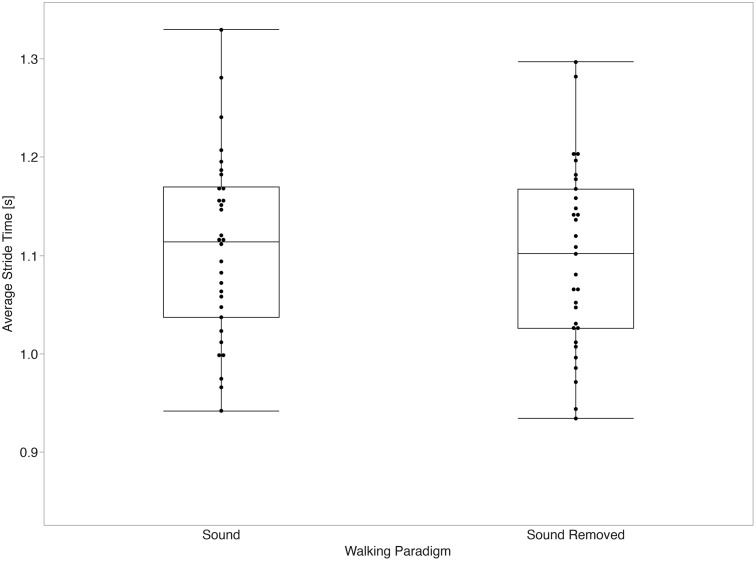
Stride time average across walking paradigms. No significant differences in stride time averages (p = 0.058) between walking paradigms were observed.

**Fig 4:**
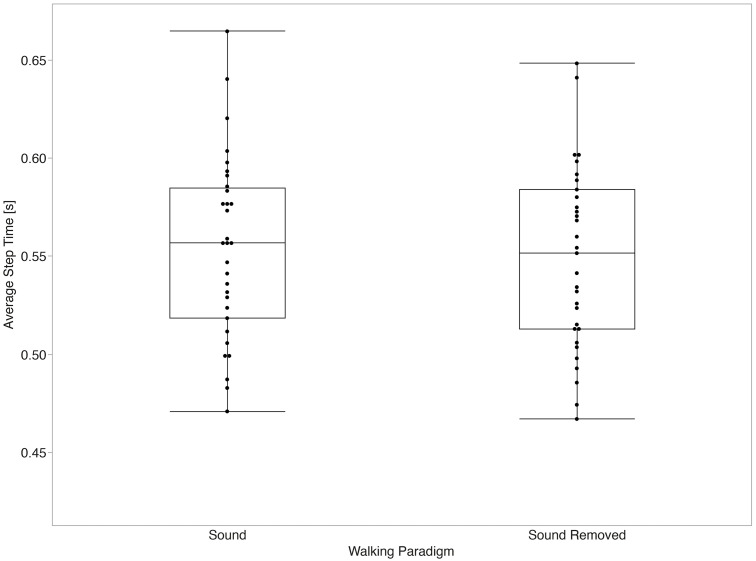
Step time average across walking paradigms. Step time was significantly longer with sound than with sound removed (p = 0.045, Cohen’s d = 0.28).

**Fig 5:**
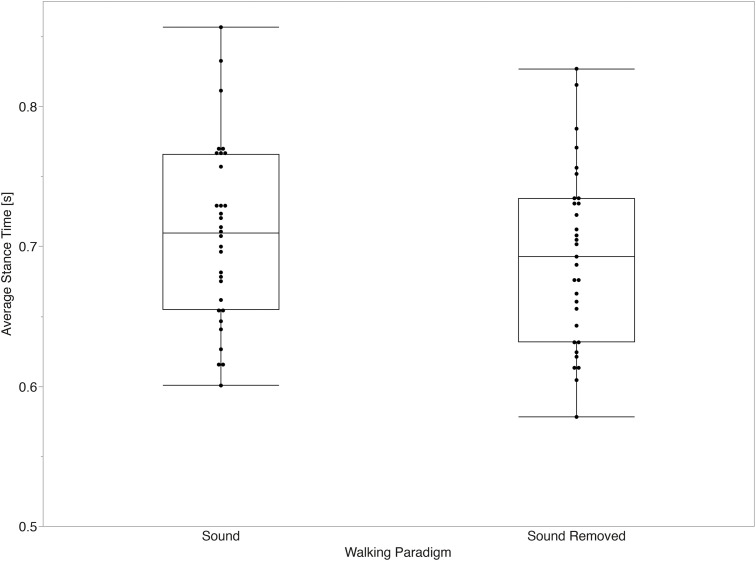
Stance time average across walking paradigms. No significant differences in stride time averages (p = 0.131) between walking paradigms were observed.

**Fig 6:**
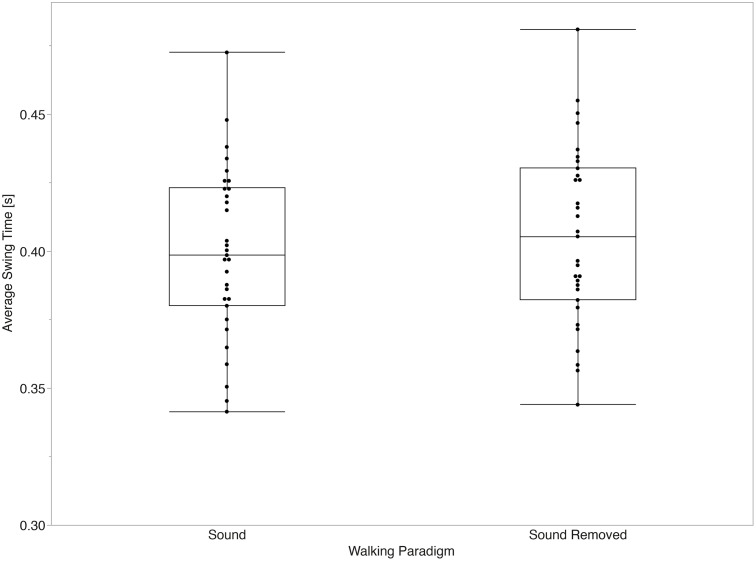
Swing time average across walking paradigms. Swing time was significantly shorter with sound than with sound removed (p < 0.001, Cohen’s d = 1.20).

The mixed effects models for average values of temporal parameters did not show a significant main effect between the sound and sound removed paradigms for stride time (p = 0.058) or stance time (p = 0.131). Significant main effects between paradigms were observed for swing time (p < 0.001, d = 1.20) and step time (p = 0.045, d = 0.28) after accounting for the effect of gait speed (all p < 0.001). Figs 3–6 summarize the comparisons of temporal average measurements of stride time, step time, swing time, and stance time between the two walking paradigms.

**Fig 7:**
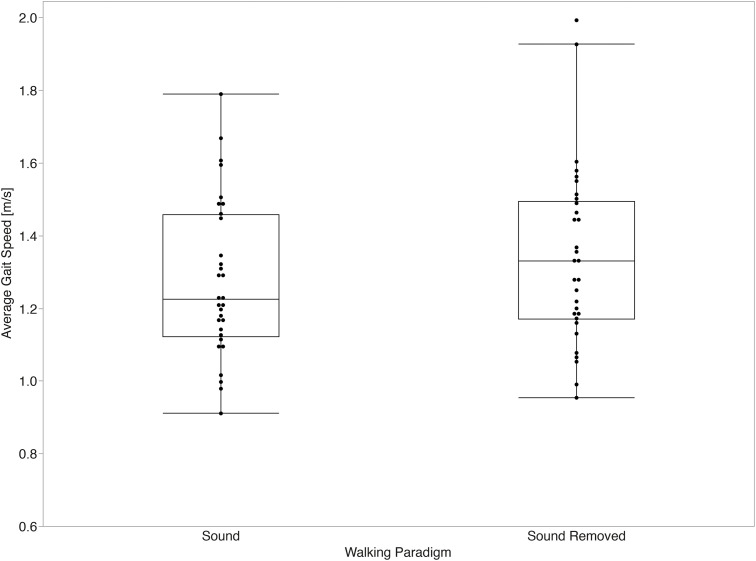
Gait speed average across walking paradigms. Walking speed was significantly faster with sound removed than with sound present (p = 0.015, Cohen’s d = 0.56).

Significant differences in average gait speed were found between walking paradigms (p = 0.015, d = 0.56). Fig 7 summarizes the comparison of average measurements of gait speed across both paradigms.

## Discussion

This study explored the effect of auditory feedback during self-pacing treadmill (SPT) walking on temporal gait parameters and gait speed. We hypothesized that there would be significant differences in average temporal walking parameters and gait speed from baseline when auditory feedback from the treadmill belt motors was removed. Both hypotheses were supported for temporal parameters, as we saw differences in temporal measurements between the baseline condition with treadmill sound and the noise cancelling plus brown noise condition that removed external auditory cues.

Significant differences were found in average swing and step time, even after controlling for walking speed. With sound present during the baseline condition, average swing times were lower and step times were higher in comparison to the test condition removing sound. This result suggests that the participants walked more cautiously to preserve stability when the treadmill sound was present. During gait, humans combine available sensory input to generate movements. One potential explanation for a careful gait is that introducing novel auditory input created either a sensory conflict or overload in response. This effect may have been amplified by the fluctuating auditory characteristics caused by the walkers’ movement along the treadmill, potentially leading to caution or apprehension while walking.

The treadmill conditions showed significant differences in average gait speed with a moderate effect size. This suggests that the noted gait speed difference is a meaningful one, i.e., gait speed is altered by the presence of auditory feedback during self-paced treadmill walking. Auditory processing has been previously reported to be an important factor in regulating locomotion speed [36], and in this study the auditory feedback signalling changed gait speed also affected walking speeds. Average gait speed was higher when the external treadmill sound was removed. During the treadmill walking, the presence of the fluctuating sound pattern could have made participants hyper-aware of their changes in speed, leading to more uneasiness and a tendency to slow their gait to maintain control.

The differences in the aforementioned gait parameters suggests that walkers on a SPT process the belt motor sounds as useful feedback to plan and initiate their movements on the treadmill. One reason that participants responded to the sound feedback may have been delays in sound feedback relative to other cues such as visual ones. Recall that when the participant walks and their estimated center of mass crosses the treadmill’s fore-aft midpoint, the controller triggers the motor to accelerate or decelerate the belts proportional to the distance of the walker’s center of mass from the treadmill’s midpoint. Given the treadmill’s inertia, there is a delay between the trigger point and the treadmill reaching its desired speed based on COM displacement. During that delay, however, the belt motors provide the auditory feedback that the belts are accelerating or decelerating. The feedback could serve two purposes: it informs the walker about the in-progress adjustment as the pitch and amplitude change, and the directionality of the sound (motors at front) could provide spatial information about their relative position, which could be important in a treadmill environment due to a reduction of visual cues which are known to be important for walking [37,38]. Delays could be a source of sensory mismatches and result in abnormal changes in gait patterns. On SPT in particular where gait speed changes don’t cause the same visual field changes as FST, walkers might upweight additional input from other senses since movement and spatial processing mandate multisensory responsibilities [39–41]. These potential differences emphasize the need for familiarization periods when walking on a self-pacing treadmill [42]. Incorporation of the sound from the motors may be even more pertinent for populations who have diminished sensory capabilities or a greater fear of falling. An additional sensory modality during walking could aid in kinesthesia and environmental awareness, alleviating the poorer quality of sensory information that can come with age or disease. More research needs to be done to fully understand the various sensory contributions and reweighting performed while walking on a SPT.

This study also presents a novel algorithm for detecting gait events from shank accelerometers on both legs. The algorithm developed here is inspired by previous work by Gurchiek et al. [35] but improves upon the errors calculated from their thigh-worn accelerometers by up to 60% (23 ms). The absolute errors found presently are comparable or less than the values reported by Gurchiek, suggesting that this method may be a more accurate estimator of gait events when walking in both treadmill and overground environments. We found errors of < 17 ms, which was about 1.5% of the gait cycle on average. This result contradicts other reports [34] that concluded that shank accelerometers were inferior placements for wearable IMUs to detect gait events, since they found errors of up to 80 ms when comparing event times to gold standard force plate data. Additionally, this study provides a thorough dataset lacking in the literature to further characterize self-pacing treadmill walking.

Several limitations exist in this study that should be considered when interpreting its results. The ordering of the trials was not randomized; the sequence was treadmill with auditory feedback, then treadmill without auditory feedback for all participants. This order was chosen because the baseline trial was established as a reference trial for comparison with other conditions. Additionally, conducting the baseline trial first allows for a ramp-up of condition difficulty, which could minimize any learning effects. The fixed ordering could introduce bias or confounding to the study, including fatigue, learning effects, or selection bias. However, since ample time is given to become accustomed to self-paced treadmill walking, we do not believe the fixed ordering should create significant biases. Sufficient time was given between conditions to avoid fatigue. Another limitation is that the two treadmill conditions compared here were not always done consecutively. Since this analysis was part of a larger walking study, there were sometimes other activities that occurred between the baseline and no sound conditions analyzed here. This intervention could possibly confound the results. To minimize this possibility we allowed at least five minutes of wash-out by walking with treadmill sounds present between every condition for the study to minimize any bias. With regard to the data collection hardware, the axes of the accelerometers used for the study did not align exactly with the outer housing of each sensor, which resulted in a slight misalignment of the accelerometers with the anatomical axes of the shanks. This discrepancy could impact the generalizability of the developed algorithm to other properly calibrated sensors. However, this discrepancy is unlikely to have affected the results since our pilot testing showed that the sensors used still reliably demonstrate the same general kinematic patterns during gait. However, it is important to note that in two participants, one of the shank sensors demonstrated acceleration waveforms with greater noise and spurious peaks, causing the gait event algorithm to fail for one leg and leading us to exclude these participants from analysis. Further research is needed to ensure high-quality data at the time of collection to support the event detection algorithm, as well as to validate the event detection algorithm in clinical populations and in daily movements other than steady-state walking.

Brown noise played through worn headphones during the sound exclusion condition was made loud enough to block the variable treadmill motor belt sounds while walking. This was effective as it blocked all environmental sounds from being heard, including the sound of footsteps that impact walking parameters [31,43], which are heightened on a treadmill due to the louder sound from frictional forces during heel strike (Supplemental Material, S1 File). In contrast, wearing headphones while walking can amplify self-generated sounds like footsteps due to occlusion effects or bone conduction and vibration. However, brown noise contains dominant low frequencies that overlap with the lower frequencies that predominate from occlusion and bone-conduction vibrations of footfalls, which serve to mask any residual low-frequency sounds due to footfalls, breathing, and other self-generated sounds. Anecdotally, this sound masking was confirmed by the authors when piloting this paradigm. One final limitation of the use of brown noise to ensure that auditory motor sounds were not heard is that brown and other colored noise may affect postural stability during quiet standing [44–47], though its isolated effects on gait are not currently known [48]. Future studies should assess potential interactions of applying brown noise while altering self-generated and environmental sounds during gait.

Finally, considerations for the exact treadmill characteristics should be taken. The instrumented treadmill used in this study was approximately 1.8 m long, so future work should explore potential effects of SPT belt length on gait patterns. Additionally, other SPT may have different sound profiles that could affect auditory perception and, ultimately, gait patterns. Further studies should also collect overground walking data to explore whether the significant differences observed in this study carry over to comparisons of walking patterns in more natural environments, and such studies should include spatial data collection to ensure the comparability of overground to treadmill parameters.

## Conclusions

Variable treadmill belt motor sounds were found to significantly affect temporal measures during walking. Participants walking on the treadmill with audible treadmill sounds and on the treadmill with all sounds removed demonstrated different gait patterns, suggesting that these sounds are used as auditory feedback for motor planning and control during treadmill walking. Additionally, a shank accelerometer-based gait event detection algorithm was developed and validated with high accuracy. The algorithm can be used for future work to study gait across treadmill and overground conditions. Further investigation into self-pacing treadmills is necessary to fully understand their differences from overground walking conditions.

## Supporting information

S1 Fig Center of mass position and belt speed during a typical trial. Belt speed is shown in the top graph and center of mass position is shown in the bottom graph. The self-pacing treadmill controller uses proportional control to keep the center of mass at −0.75m (green line), near the center of the treadmill in the anterior-posterior direction.
(PDF)

S1 File Audible feedback, belt speed, and center of mass position during a typical trial. In the sound present walking paradigm, participants can hear both the treadmill motors and the sound of the belt passing over the treadmill deck. Both amplitude and frequency of the audible sounds provide continuous feedback to participants of their current walking speed. The vertical red lines of the video show the current time, demonstrating the changes in frequency that are proportional to the walking speed.
(MP4)

S2 File TreadmillSoundsData. This file archive contains gait events from the two test conditions (no sound and with sound) for all included participants, as well as the validation data for the accelerometer-based gait event detection algorithm. All data files are in CSV format. A readme file is included with the details of the file format and contents.
(ZIP)
