# Supplementary figures and images for: Auditory feedback effect on temporal patterns during self-pacing treadmill walking
Source: PLoS One. 2025 Nov 4;20(11):e0335971. doi: 10.1371/journal.pone.0335971 (PMC12585087; doi:10.1371/journal.pone.0335971)

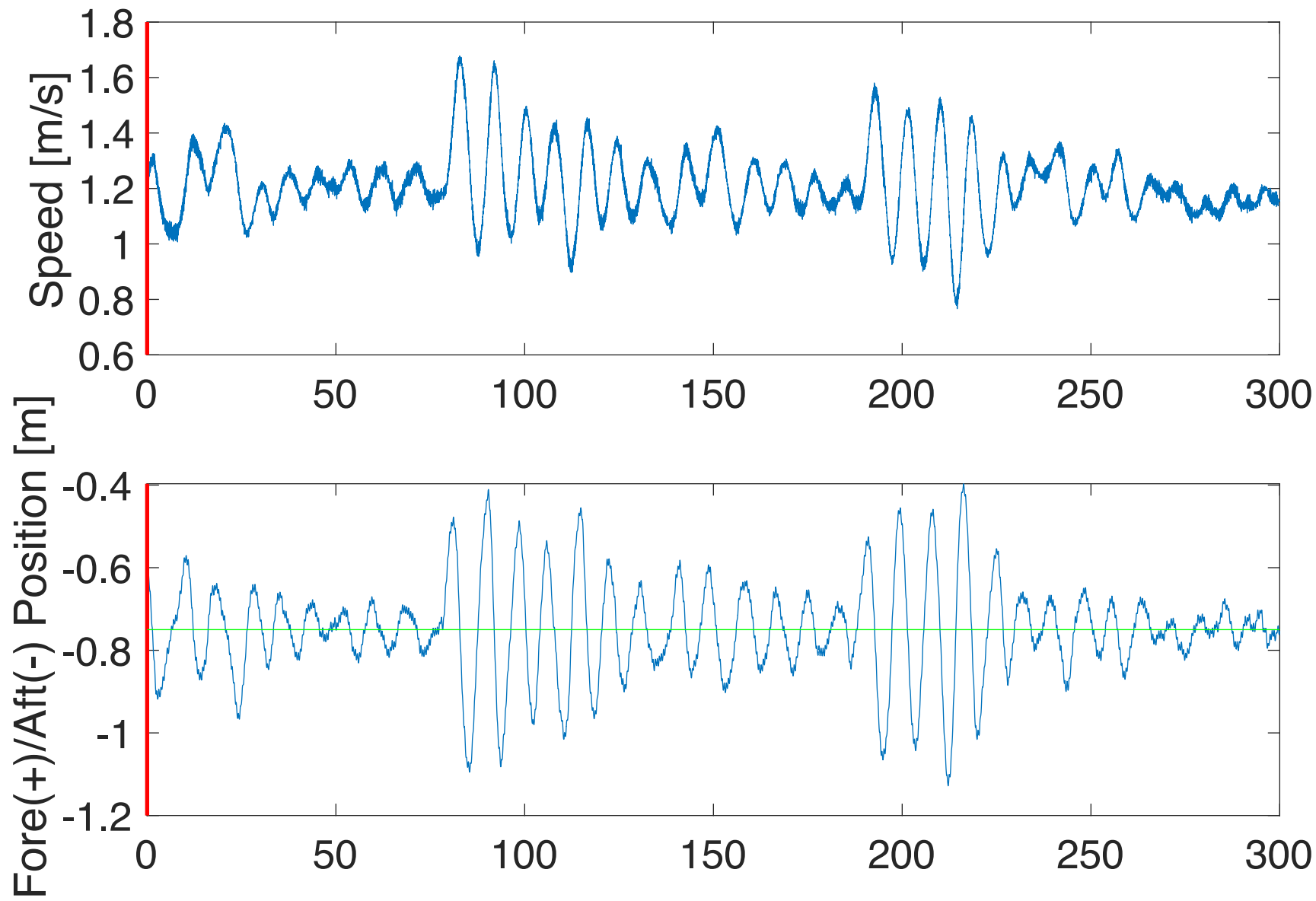

Supplement: S1 Fig — Belt speed is shown in the top graph and center of mass position is shown in the bottom graph. The self-pacing treadmill controller uses proportional control to keep the center of mass at −0.75m (green line), near the center of the treadmill in the anterior-posterior direction. (PDF) [file pone.0335971.s001.pdf]
